# Supplementary material for: Knowledge, attitude and practice of wearing mask in the population presenting to tertiary hospitals in a developing country
Source: PLoS One. 2022 Mar 10;17(3):e0265328. doi: 10.1371/journal.pone.0265328 (PMC8912125; doi:10.1371/journal.pone.0265328)
Supplement: S1 Questionnaire — (PDF) [file pone.0265328.s002.pdf]

PROFORMA

Patient Details

Case #:	________________________	
Age (years):	________________________	
**Gender**:	☐ Male	☐ Female
**Residence status**:	☐ Rural	☐ Urban
**Socioeconomic class:**	☐ Low (≤ 25000 per month)	
	☐ Middle (25000-50000 per month)	
	☐ High (≥ 50000 per month)	
**Education:**	☐ Illiterate (Never went to school)	
	☐ Primary (Class1-5)	
	☐ Secondary (Class 6-Matric)	
	☐ Higher (Intermediate to graduation)	
Family COVID-19 history:	☐ Yes	☐ No
Exposed to COVID-19 patients:	☐ Yes	☐ No

Co-morbid Conditions

Hypertension Treatment?	☐ Yes	☐ No
Diabetes?	☐ Yes	☐ No
Smoking?	☐ Yes	☐ No
If Yes;	☐ Current Smoker	☐ Ex-smoker
Asthma/COPD?	☐ Yes	☐ No
Ischemic heart disease?	☐ Yes	☐ No

Practice of Using Face Mask

	Yes<br>ہاں	No<br>نہیں	
Did you wear mask in your day to day life during COVID-19 pandemic?	☐	☐	کیاآپ نے کورونا کے وباء کے دوران اپنے روزمرہ کی زندگی میں ماسک پہن رکھا ہے؟
If yes; please tell us about your mask wearing practice specific to the following situations;			اگر ہاں؛ براہ کرم ہمیں مندرجہ ذیل حالات میں اپنے ماسک پہننے کی عادت کے بارے میں بتائیں۔

Situation	Never<br>کبھی نہیں	Sometimes<br>کبھی کبھار	Always<br>ہمیشہ	NA<br>قابل اطلاق نہیں	حالات
When taking care of family members with fever	☐	☐	☐	☐	بخار میں مبتلا خاندان کے کسی فرد کی دیکھ بھال کرتے ہوئے۔
When taking care of family members with respiratory infection	☐	☐	☐	☐	سانس کے انفیکشن میں مبتلا خاندان کے کسی فرد کی دیکھ بھال کرتے ہوئے۔
When visiting clinics during peak season or a flu pandemic	☐	☐	☐	☐	وباء کے عروج دوران کلینک کا دورہ کرتے ہوئے۔
When visiting hospitals during peak season or a flu pandemic	☐	☐	☐	☐	وباء کے عروج دوران ہسپتال کا دورہ کرتے ہوئے۔
When having respiratory symptoms	☐	☐	☐	☐	جب کبھی سانس لینے میں دشواری جیسی علامات ظاہر ہوں۔

Technique of Using Face Mask

For all the participants who said "yes" to face mask wearing;<br>Please tell us about steps you follow while wearing face mask?;	ان شرکاء سے سوالات پوچھیں جو مارسک کا استعمال کرتے ہیں۔<br>براہ کرم اپنے ماسک پہننے کے طریقہ کار کے بارے میں بتائیں؟

Steps	Yes	No	طریقہ کار
	ہاں	نہیں	

**Putting on**			**ماسک لگانا**
Perform hand hygiene before wearing the face mask	☐	☐	چہرے پر ماسک لگانے سے پہلے ہاتھوں کی صفائی کرتے ہیں۔
Choose the appropriate size of face mask	☐	☐	چہرے کے مطابق ماسک کا مناسب سائز منتخب کرتے ہیں۔
Ensure the colored side of the face mask is facing outwards	☐	☐	اس بات کو یقینی بناتے ہیں کہ ماسک کی رنگین سائیڈ باہر کی طرف ہو۔
Position the elastic band properly	☐	☐	لچکدار بینڈ کو مناسب طریقے سے کرتے ہیں۔
Press firmly on the metallic strip to the bridge of nose and face	☐	☐	ناک پر لگی دھاتی پٹی پر مضبوطی سے دباتے ہیں۔
Extend the face mask to cover mouth, nose and chin	☐	☐	منہ ، ناک اور ٹھوڑی کو ڈھانپنے کے لئے ماسک کو بڑھاتے ہیں۔
Avoid touching the face mask once it is secured	☐	☐	ایک بار پہننے کے بعد ماسک کو چھونے سے گریز کرتے ہیں۔
**Taking off**			**ماسک اتارنا**
Perform hand hygiene before taking off the face mask	☐	☐	چہرے سے ماسک اتارنے سے پہلے ہاتھوں کی صفائی کرتے ہیں۔
Touch only the elastic bands	☐	☐	صرف لچکدار بینڈ کو چھوتے ہیں۔
Dispose of the used face mask in a lidded rubbish bin	☐	☐	استعمال شدہ ماسک کو ڈھکنے والے کوڑے دانوں میں ڈال تے ہیں۔
Perform hand hygiene after disposing the face mask	☐	☐	ماسک کو ضائع کرنے کے بعد ہاتھوں کی صفائی کرتے ہیں۔
